# Developing strategic priorities in osteoarthritis research: Proceedings and recommendations arising from the 2017 Australian Osteoarthritis Summit

**DOI:** 10.1186/s12891-019-2455-x

**Published:** 2019-02-13

**Authors:** David J. Hunter, Philippa J. A. Nicolson, Christopher B. Little, Sarah R. Robbins, Xia Wang, Kim L. Bennell

**Affiliations:** 1 Department of Rheumatology, Royal North Shore Hospital and Institute of Bone and Joint Research, Kolling Institute, University of Sydney, St Leonards, Sydney, New South Wales 2065 Australia; 2 Centre for Health, Exercise and Sports Medicine, Department of Physiotherapy, University of Melbourne, Melbourne, Australia; 3 Raymond Purves Bone and Joint Research Laboratories, Royal North Shore Hospital, Kolling Institute and Institute of Bone and Joint Research, University of Sydney, Sydney, Australia; 4 Rheumatology Department, Royal North Shore Hospital, Reserve Road, St Leonards, New South Wales 2065 Australia

**Keywords:** Osteoarthritis, Research priorities

## Abstract

**Background:**

There is a pressing need to enhance osteoarthritis (OA) research to find ways of alleviating its enormous individual and societal impact due to the high prevalence, associated disability, and extensive costs.

**Methods:**

Potential research priorities and initial rankings were pre-identified via surveys and the 1000Minds process by OA consumers and the research community. The OA Summit was held to decide key research priorities that match the strengths and expertise of the Australian OA research community and align with the needs of consumers. Facilitated breakout sessions were conducted to identify initiatives and strategies to advance OA research into agreed priority areas, and foster collaboration in OA research by forming research networks.

**Results:**

From the pre-Summit activities, the three research priority areas identified were: treatment adherence and behaviour change, disease modification, and prevention of OA. Eighty-five Australian and international leading OA experts participated in the Summit, including specialists, allied health practitioners, researchers from all states of Australia representing both universities and medical research institutes; representatives from Arthritis Australia, health insurers; and persons living with OA. Through the presentations and discussions during the Summit, there was a broad consensus on the OA research priorities across stakeholders and how these can be supported across government, industry, service providers and consumers.

**Conclusion:**

The Australian OA Summit brought consumers, experts and opinion leaders together to identify OA research priorities, to enhance current research efforts by fostering collaboration that offer the greatest potential for alleviating the disease burden.

**Electronic supplementary material:**

The online version of this article (10.1186/s12891-019-2455-x) contains supplementary material, which is available to authorized users.

## Background

Osteoarthritis (OA) is a highly prevalent, disabling condition that affects nearly three million, or one in 11, Australians [1]. The risk of mobility disability (defined as needing help walking or climbing stairs) attributable to knee OA alone is greater than that due to any other medical condition in people aged 65 and over [2, 3]. OA costs the Australian health system over $2 billion, and the economy over $23 billion annually [4]. The prevalence of OA is expected to double to one in four Australians by 2040, due to an ageing and increasingly obese population [5].

OA is poorly managed in Australia. Two-thirds of people with OA report that they are faring badly with their condition [6], 57% do not receive appropriate care according to current guidelines [7], and most general practitioners (GPs) report dissatisfaction with the care they can provide to people with OA due to the limited effectiveness of current OA treatment options [8]. Management is compromised by the fact that little is known about the causes of OA and there is no confirmed cure or intervention to slow its progression.

Even with the increasing disease burden and the pressing need for cost-effective management in OA, the current research funding in this field is insufficient and fragmented [9]. Together with other musculoskeletal disorders, OA has been recognised as a National Health Priority Area, but National Health and Medical Research Council (NHMRC) funding for OA research was only $8 million in 2017 [10], which is less than 0.35% of the health system costs of managing the disease. This level of research funding is substantially lower than many other similar chronic conditions. Take the example of diabetes research, there were $74.9 million funded by NHMRC in 2017, which was equal to 5.6% of the healthcare expenditure (~$1.1 billion) of the condition [10].

In addition, research in OA in Australia tends to be disjointed and is mainly conducted by isolated and disparate research groups. The Australian OA research community has tremendous strengths in both basic and clinical research that are internationally recognised. Enhancing collaborations between researchers in these two areas will be important to fully realise the capacity of OA research in Australia.

The 5th Australian OA Research Summit was held on May 30th, 2017 at the Kolling Institute, Royal North Shore Hospital, Sydney. The Summit was intended as a further step in an ongoing strategic planning process to enhance OA research in Australia. Through the process, key research priorities were recognised that not only match the strengths and expertise of the Australian OA research community, but also align with the areas of greatest interest identified by people with the disease. The major aims for the day were to:

- identify research priorities for the next three to 5 years that offer the greatest potential benefit for alleviating the burden of OA for patients in Australia;
- identify initiatives and strategies to advance OA research into agreed priority areas;
- foster increased collaboration in the Australian OA research community by facilitating the formation of research groups and networks to increase investigative power.

The Summit provided an opportunity to highlight the strengths, expertise and breadth of Australian OA researchers. In addition, it emphasised to the major Australian funding bodies the enormous disparity between the burden of OA disease and the level of research funding in Australia. The Summit also afforded an opportunity for multidisciplinary networking and collaboration between participants. Summit participants comprised leading Australian and international OA experts, researchers and opinion leaders, including rheumatologists, surgeons, physiotherapists and research scientists, as well as research funders, healthcare providers, insurers and consumers. This paper provides a summary of the proceedings and outcomes of the OA Summit with the aim of developing a broad consensus on OA research priorities in Australia, and how these can be supported across government, industry, funding organisations, researchers, health practitioners, service providers and consumers.

## Methods

### Process

The priority setting process comprised four main stages:

- Stage 1: Pre-identification of potential research priorities via online surveys before the Summit.
- Stage 2: Initial ranking of suggested research priorities using the 1000Minds process before the Summit (see Box 1), firstly, by people with OA and secondly, by the OA research community.
- Stage 3: Identification of potential thematic research priorities
- Stage 4: Discussion and presentation of thematic priorities at the Summit

### Participants

Stages 1 and 2 were completed separately among persons with OA, and among OA Summit registrants or confirmed delegates.

People with OA were recruited via two methods: i) advertisements placed on social media, and ii) from the databases of previous volunteers who had consented to be contacted for further studies from the University of Melbourne. The advertisement contained links to access further information about the study and to complete the questionnaire online. To be eligible to take part, respondents were required to self-report OA of one or more joints, and be able to understand and read English. People with OA identified from existing databases were emailed a letter and link to the survey.

The Australian OA Summit brought together 85 Australian and international leading OA experts including rheumatologists (*N* = 8); orthopaedic surgeons (*N* = 4); allied health practitioners (*N* = 17); clinical, epidemiological and basic science researchers (*N* = 22); representatives from non-for-profit organisations (*N* = 5) and industry (N = 17); and persons living with OA (*N* = 6).

Leading international OA researchers and opinion leaders, Professors Stefan Lohmander (Lund University, Sweden), Richard Loeser (University of North Carolina, USA), and Philip Conaghan (Leeds University, UK), were invited to participate in the Summit. These world leaders brought their combined expertise in orthopaedics, basic science and rheumatology, to provide a valuable, independent international perspective on Australian OA research, as well as examples of OA research prioritisation and international coordination.

The participants invited to attend were considered to be national opinion leaders based on the knowledge of the Summit Program Committee, evaluation of NHMRC and Australian Research Council (ARC) grants awarded in the past 5 years, and a review of the literature in PubMed using the search terms “osteoarthritis” and “Australia”. Upon invitation, each invitee was asked to recommend colleagues who should also be invited.

At the time of registration for the OA Summit, attendees were advised of Stages 1 and 2 and invited to take part. All participants gave implied consent by completion of the questionnaire, and subsequently the pairwise ranking survey.

#### Stage 1: Pre-identification of potential research priorities

Participants completed Stage 1 online using two survey software: SurveyGizmo and REDCap. The survey for people with OA comprised two sections: the first section contained basic demographic questions such as gender and age, as well as questions related to their OA pain, predominant joint with OA, length of time experiencing symptoms and any joint replacements. In the second section, respondents were asked to list in their own words what they considered to be the top 3 research priorities related to OA. For OA Summit attendees, the survey only contained the second section.

Data were downloaded and imported into an excel database. Research suggestions were compiled and analysed by three authors (PJAN, KLB, DJH) to determine common themes as topics for Stage 2.

#### Stage 2: Priority setting method

Prioritisation of research topics was achieved using 1000Minds (see below for details) [11]. Separate surveys were built for people with OA and Summit attendees, using the most commonly suggested research topics from Stage 1. The link to the online survey was emailed to respondents from Stage 1. In 1000Minds, respondents were shown two research priorities and asked to identify which they believe should be more of a priority. These two alternative sets are known as ‘pair-wise rankings’. The number of pairwise rankings each respondent was required to make varied depending on how each answered the set of alternatives presented. The pairwise ranking continued until the background mathematics was able to establish a ranked list of all items for each respondent.

### Description of 1000Minds program

This program (www.1000Minds.com) developed at the University of Otago, New Zealand. It is a multi-attribute decision analysis research tool that prioritises and quantifies the relative importance of criteria, based on expert preferences. The central question in each theme was: “Which of these (two) research areas would you consider having the highest priority in the Australian setting?”

1000Minds was chosen because it is more user-friendly than alternate consensus or prioritisation processes; it requires decisions on a series of pairs (alternate scenarios) only, rather than ranking multiple alternatives at one time. The 1000Minds process also eliminates the “loudest voice” in a consensus process. Analysis of participants’ choices occurs in the background so that group results are processed automatically and quickly regardless of how many participants are included in the task.

#### Stage 3: Identification of potential thematic research priorities

Before the Summit, the Summit Program Committee reviewed the results from Stages 1 and 2, and by iterative discussion identified three key themes for the priority setting process that broadly aligned with the top priorities from both consumers and OA Summit attendees.

#### Stage 4: Discussion and presentation of thematic priorities

All attendees were randomly assigned to one of four breakout groups on the day of the Summit. The objective of these breakout sessions was to refine the research priorities identified and ranked in the 1000Minds exercise, and flesh out some suggested approaches to pursuing these priorities.

Discussions in the four breakout groups were moderated and recorded by two group leaders who were pre-selected from the list of participants. The groups were given the task of developing an integrated PROGRAM of research for each theme. These programs were to involve a cross-disciplinary approach (e.g. clinical, health delivery, basic science, maybe also academia/health/industry etc.). The idea was to also identify the pillars or individual projects within each theme and cross-cutting concepts/themes. Each group was asked to complete a template for each of the three research priorities identified by the group covering: the research priority title or aim; a précis of methods to address the question; and consideration of existing strengths in the area within Australia. The ranking of research questions from the 1000Minds exercise was presented to all Summit attendees before and again at the meeting.

Following the breakout group discussions, the discussion leaders for each group presented their plans to all Summit participants. The individual topic or priority areas were then opened for general discussion and questions by all Summit participants. Following the Summit, the three thematic areas and the breakout group discussions were collated and further refined.

## Results

Table 1 outlines the demographic characteristics of respondents with OA (*N* = 161). The group were an average 67 years of age, 64% were female, 78% had OA in multiple joints and the average disease duration was 12 years.

**Table 1:** Demographic characteristics of respondents with OA (*n* = 161)

	Mean ± SD or n (%)
Age (years)	66.7 ± 6.8
Female sex (n (%))	103 (64)
State / Territory of Australia	
Victoria	78 (48)
Queensland	38 (24)
New South Wales	22 (14)
Tasmania	7 (4)
South Australia	6 (4)
Australian Capital Territory	5 (3)
Western Australia	4 (2)
Northern Territory	1 (1)
Symptom duration (years)	11.9 ± 10.5
Multi-joint osteoarthritis (*n* (%))	127 (78)
Right foot	22 (14)
Left foot	20 (12)
Right ankle	17 (10)
Left ankle	13 (8)
Right knee	103 (64)
Left knee	89 (55)
Right hip	64 (40)
Left hip	48 (30)
Lumbar spine	49 (30)
Thoracic spine	14 (9)
Cervical spine	29 (18)
Right shoulder	21 (13)
Left shoulder	14 (9)
Right wrist	16 (10)
Left wrist	15 (9)
Right hand	38 (23)
Left hand	36 (22)
Consulted a health practitioner about OA (*n* (%))	151 (93)
General practitioner	136 (84)
Physiotherapist	100 (62)
Orthopaedic surgeon	88 (54)
Exercise physiologist	38 (23)
Chiropractor	30 (19)
Rheumatologist	22 (14)
Podiatrist	19 (12)
Osteopath	18 (11)
Sports physician	14 (9)
Dietitian	13 (8)
Occupational therapist	6 (4)
Pain specialist	6 (4)
Had surgical intervention for OA (*n* (%))	64 (40)
Knee arthroscopy	33 (20)
Total hip replacement	20 (13)
Total knee replacement	17 (11)
Hip arthroscopy	3 (2)
Ankle arthrodesis	3 (2)
Lumbar laminectomy	2 (1)
Shoulder arthroscopy	1 (0.6)
Total shoulder replacement	1 (0.6)
Partial knee replacement	1 (0.6)
High tibial osteotomy	1 (0.6)
Talo-navicular fusion	1 (0.6)
1st metatarsal phalangeal joint fusion	1 (0.6)

SD = standard deviation; *n* = number

Tables 2 and 3 summarise the final group results following the 1000Minds process for the persons with OA and OA Summit attendees respectively. The three key themes of the priority setting process that broadly aligned with the top priorities from both the consumers and OA Summit attendees were:

1. Treatment adherence and behaviour change
2. Disease modification or modifying progression
3. Prevention of OA

**Table 2:** Prioritised rankings of research topics by persons with OA (1000Minds survey responders *n* = 161)

Ranking	Topic	Median	Mean	Q1	Q3	IQR
1	Preventing further progression of osteoarthritis once identified	4.50	5.49	3.00	8.00	5.00
2	Prevention of osteoarthritis	5.00	6.08	2.50	9.00	6.50
3	Identifying the cause of osteoarthritis	5.50	6.40	2.50	9.25	6.75
4	A cure for osteoarthritis	5.50	6.36	2.00	8.75	6.75
5	Stem cell / Platelet rich plasma treatments	7.00	7.90	3.00	12.00	9.00
6	Identifying people with early osteoarthritis	8.00	8.57	4.00	12.00	8.00
7	Non-surgical treatments for osteoarthritis	8.00	7.92	5.00	10.50	5.50
8	Identifying triggers for pain	9.00	9.19	6.00	12.50	6.50
9	Identifying the role of genetics	10.00	10.03	5.50	14.00	8.50
10	Exercise	10.50	10.80	7.00	14.75	7.75
11	Cartilage replacement	11.00	10.60	6.00	16.00	10.00
12	Physiotherapy	11.00	11.11	8.00	14.50	6.50
13	Improving education about osteoarthritis	12.50	11.90	8.00	15.75	7.75
14	Diet and weight loss treatments	13.00	11.49	7.50	15.50	8.00
15	Alternative therapies / supplements	13.00	12.37	8.00	16.75	8.75
16	Optimising pain medications	13.50	12.49	9.50	16.00	6.50
17	Surgical treatments	14.50	13.87	11.00	18.00	7.00
18	Therapeutic aids / assistive devices	14.50	13.54	11.00	16.50	5.50
19	Footwear	15.00	13.88	11.00	18.00	7.00

The ranking numbers represent the ordinal ranking of the priority values. For example 1 = 1st, i.e. this is the top ranking or top test for the group. The median/mean represents the group median/mean ranking with 1st (Q1) and 3rd (Q3) quartiles and interquartile range (IQR)

**Table 3:** Prioritised rankings of research topics by OA summit attendees (1000Minds survey responders *n* = 58)

Ranking	Topic	Median	Mean	Q1	Q3	IQR
1	Treatment adherence and behaviour change	5.50	6.37	3.50	8.00	4.50
2	Disease modification / preventing progression	6.00	7.20	4.00	11.00	7.00
3	Targeted- personalised treatment	6.50	7.09	3.50	11.00	7.50
4	Prevention of osteoarthritis	7.00	7.74	4.00	11.50	7.50
5	Non-drug, non-surgical interventions	7.50	7.96	4.50	11.00	6.50
6	Implementation / models of service delivery	8.00	8.39	3.00	13.00	10.00
7	Self-management	8.00	8.35	5.00	13.00	8.00
8	Diet and weight loss	9.50	9.22	5.00	14.00	9.00
9	Mechanisms of disease and treatments	10.00	9.48	5.50	13.00	7.50
10	Exercise	10.00	9.81	5.00	15.00	10.00
11	Use of technology in delivering treatments	10.50	10.59	6.00	16.50	10.50
12	Pain management	10.50	10.17	6.00	13.50	7.50
13	Sub-grouping / phenotyping	10.50	10.65	5.00	16.50	11.50
14	Selection of optimal patients for surgery	11.50	10.93	7.00	15.00	8.00
15	Early identification / biomarkers	12.50	11.66	8.00	15.00	7.00
16	Optimising surgical outcomes	14.00	11.87	6.00	17.00	11.00
17	PRP/ Stem cells / Cartilage repair	15.00	13.50	8.00	18.50	10.50
18	Pre-clinical models	16.00	13.87	10.00	17.50	7.50
19	Medications	16.00	15.15	13.00	18.00	5.00

The ranking numbers represent the ordinal ranking of the priority values. For example 1 = 1st, i.e. this is the top ranking or top test for the group. The median/mean represents the group median/mean ranking with 1st (Q1) and 3rd (Q3) quartiles and interquartile range (IQR)

Tables outlining the collated key points of discussion for each of the three research priorities are included as an additional file 1, with a narrative summary of each thematic priority below.

### Research priority 1: Treatment adherence and behaviour change

Treatment guidelines for OA recommend non-drug, non-surgical treatment as the cornerstone of management, particularly interventions that foster appropriate lifestyle behavioural change such as patient education, exercise and if applicable, weight loss. However, there is evidence that care for people with OA is suboptimal in Australia. Clinician factors relating to suboptimal care involve under-use of these core treatments, lack of or inappropriate referral by GPs to allied health professionals, an overwhelming reliance on drugs or surgery, and use of a “biomedical” approach that does not facilitate patient-centred care nor patient behaviour change. Patient-related factors include misconceptions about their OA condition, assessment and treatment. Patients also lack confidence to participate in decision-making about their treatment options, and there is lack of uptake and adherence to treatments particularly exercise and weight loss.

A number of aims in this research priority were discussed by the breakout group and wider Summit audience. These included:

- providing evidence to show that better adherence to treatment translates into improved outcomes
- identifying barriers and enablers to adherence to exercise and weight loss interventions
- identifying responders and non-responders to treatment to match patient phenotype to appropriate effective treatment
- examining whether it is possible to predict adherence to treatment and define a risk level whereby additional adherence strategies would be implemented
- comparing the effectiveness of treatment programs to improve adherence and outcomes for patients
- focusing on long-term maintenance of lifestyle behaviour change post OA programs and sustainability of outcomes
- testing different education delivery modes to both consumers and clinicians
- investigating the effectiveness of strategies to improve adherence to guidelines by clinicians

While detailed research methods were not specifically discussed, several aspects were mentioned. One was the importance of observational studies leading to trials as well as qualitative research to understand factors influencing implementation, contextual adaptation and sustainability. Randomized controlled trials (RCTs) including cluster and pragmatic designs and involving multi-sites and multi-disciplines were highlighted. Stepped approaches were also discussed whereby patients are treated with different interventions depending on their adherence and response to earlier treatments. There may also be potential for private providers to routinely collect useful data that could be pooled and analysed.

It was noted by the group that many OA treatment programs are being rolled out nationally but these programs are not necessarily being rigorously evaluated or monitored. These programs provide an opportunity for better understanding of barriers, enablers and outcomes (both clinical and financial) in the real-world setting. Other perceived opportunities and potential funding sources includeAustralia & New Zealand Musculoskeletal Clinical Trial (ANZMUSC) which can help facilitate multi-site clinical trials, local health district networks, health insurers, the Health departments, the Medical Research Future Fund (MRFF) and NHMRC partnership grants.

### Research priority 2: Disease modification or modifying progression

OA is increasingly being recognised as a disease affecting all the different tissues in the joint, extra-articular tissues such as skeletal muscle and tendon, regulated by and feeding back to systemic factors including metabolic state, and the inflammatory/immune system. This complex interaction regulates not just the structural disease and its progression, but activation of local and central pain recognition and regulation pathways. This led to a considerable discussion about the critical need to have standardised definitions of OA that recognise and measure all aspects of the disease at both the local and systemic level.

A number of aims in this research priority were discussed by the breakout group and wider Summit audience. These included:

- disease progression and its measurement must be clearly defined in terms of symptoms or pathology or both, in order to understand the interaction and mutual effects among structure, function and pain, and to predict characteristics of OA over time
- defining different OA phenotypes that differ in progression and/or response to therapy is vital for clinical trial design and treatment implementation
- identifying the clinical trajectories (symptoms or disability) of people with OA and the risk factors for each of these trajectories, including characterising flares and persistence
- identifying biomarkers of structural progression that are useful and predictive within the temporal constraints of typically funded trials
- emphasising the “whole of patient” disease, not just the local joint OA, and the need to collect whole patient data versus isolated aspects to improve studies, e.g. within-subject studies show a good correlation between pathology and symptoms in different joints that is not evident when comparing joints between patients
- engaging bona fide pain researchers that understand genomics, molecular and psychological regulation of symptoms
- developing or validating animal and laboratory pre-clinical models that are more predictive of human disease pathology and symptoms is essential for therapeutic development
- defining the role of weight loss and/or exercise in modifying disease progression: maintenance of weight loss and “treat to target” strategies (e.g. achieve certain weight loss by whatever it takes)

Research methods were not delineated in detail, but several key fundamentals were noted. Multi-centre, multi-disciplinary, multi-platform studies to enable whole patient outcomes were emphasised. This concept included inviting cardiologists, diabetologists etc. to participate, and linking with state and national public health campaigns, “healthy eating”, and school programs. Optimal outcomes would be achieved by clinical trialists collaborating with basic scientists and pain researchers/experts to understand phenotypes and inform outcomes. RCTs including clustering, e.g. by phenotype, sporting teams etc. were highlighted. A perceived strength was the suitability of the research environment in Australia to study implementation, through the presence of international expertise across OA basic science, pre-clinical models, pain, clinical trialists, and ANZMUSC to ensure optimal trial design and coordination. Potential funding opportunities included NHMRC, MRFF (clinical trial call in late 2017), industry for both structural and symptomatic therapeutics, and ensuring we tap into agencies traditionally focused on other targets but where OA is a major contributor or co-morbidity, e.g. obesity, diabetes, pain.

### Research priority 3: Disease prevention

OA is a slowly progressive disease that often takes decades to develop. The two major risk factors for its development: overweight or obesity and joint injury are both modifiable [12]. Prevention strategies are available to reduce OA incidence in our increasingly aged population with increasing rates of joint injury and obesity in our community.

A number of aims in this research priority were discussed by the breakout group and wider OA Summit audience. These included:

- primary prevention opportunities through weight loss or injury prevention
- secondary prevention opportunities of targeting those with a knee injury to prevent further progression
- consideration of refining modifiable triggers to prevent disease flares and prevent acute nociceptive pain progressing into more complex chronic pain

Research methods were not delineated in detail, but several key fundamentals were noted. These included the consideration of the development of public health campaigns enlisting the support of famous sports people. Suggestions of clustering by sport and testing different joint injury prevention programs in different sports. Potential funding opportunities included applying to health insurance or accident insurance companies.

## Discussion

A key message from the OA Summit was the importance of promoting and supporting effective management of OA at all stages of the condition, rather than the current emphasis on palliation as the disease progresses. This is reflected in the top research priorities including prevention and disease modification.

Several international efforts have been made in prioritising OA research by different OA initiatives and specialty groups, which are largely aligned with the priorities identified by the Summit. The Osteoarthritis Research Society International-US Food and Drug Administration (OARSI-FDA) initiative [13], the Outcome Measures in Rheumatology (OMERACT) hand OA research priorities [14] and Patient-centred Outcomes Research Institute [15] have recognised the common domains on the topic of OA research priorities including the need for better patient centred outcome measures, methods to predict progression and define early OA or disease phenotypes. These are consistent with our priorities in promoting patient adherence and preventing progression. In addition, the European League Against Rheumatology (EULAR) expert committee identified strategic prioritisation focusing on epidemiology, pathogenesis, imaging and biomarkers and therapy [16]. Similar priority themes which were identified by both EULAR and our Summit include predictors of progression, mechanisms of pain, and individualised or optimal combination therapy strategies. Other recommendations emphasising the need for improving biomarkers (imaging and biochemical), knowledge of OA pathophysiology (tissue communication, pain and structure), and early intervention in post-traumatic OA, while extensively discussed during our Summit have not been prioritised taking into account Australian circumstance.

The priorities from the OA Summit reflect the composition of people with OA and Summit attendee groups that volunteered to participate and completed the 1000Minds exercise. While all potentially affected joints were present in the people with OA, the prevalence of hand OA may be under-represented based on known population prevalence data [17, 18]. Although the survey results show that 78% of people reported having OA at multiple joints, which are also likely to have hand joints involvement. In the absence of exact data, it is unclear if this might affect the outcome of priority setting, for example research questions related to prevention and management of joint injury might be more relevant to those with knee and ankle OA compared with hand. Therefore, a broader consultation with the OA research community, and other stakeholders will be required to build a consensus on the final research priorities. It is of course important that such strategy setting should never exclude novel ideas that may move our understanding or treatment of OA forward.

### Next steps

In addition to broader consultation on the priorities identified at the Summit, potential next steps identified by Summit attendees included:

1. Establish an OA research network to support ongoing priority setting, collaboration and coordination of research effort.
2. Leverage funding for further research support by discussing with current funding partners the opportunity to collegially support these thematic research priorities.
3. Utilise this momentum and conduct an environmental scan to feed into a national OA strategy. The strategy would make best practice OA management accessible to all Australians through harnessing the expertise of all stakeholders including health professionals, private sector partners, industry, relevant not-for-profit organisations, consumer groups, non-government payers (including general, non-health insurers) and state and federal governments.

## Conclusion

The Summit was intended as a starting point in an ongoing strategic framework to identify major research priorities that match the strengths and expertise of the Australian OA research community and offer the greatest potential benefit for alleviating the burden of OA.

## Additional file

Additional file 1: Thematic discussions. Meeting notes in break-out group discussions. (DOCX 22 kb)

## Abbreviations

ANZMUSC: Australia & New Zealand Musculoskeletal Clinical Trials Network
ARC: Australian Research Council
EULAR: European League Against Rheumatology
FDA: Food and drug administration
GPs: General practitioners
MRFF: Medical Research Future Fund
NHMRC: National Health and Medical Research Council
OA: Osteoarthritis
OARSI: Osteoarthritis research society international
OMERACT: Outcome Measures in Rheumatology
RCTs: Randomized controlled trials
