# Supplementary material for: Developing strategic priorities in osteoarthritis research: Proceedings and recommendations arising from the 2017 Australian Osteoarthritis Summit
Source: BMC Musculoskelet Disord. 2019 Feb 13;20:74. doi: 10.1186/s12891-019-2455-x (PMC6375218; doi:10.1186/s12891-019-2455-x)
Supplement: Supplementary file 1 — Thematic discussions. Meeting notes in break-out group discussions. (DOCX 22 kb) [file 12891_2019_2455_MOESM1_ESM.docx]

**Additional file 1: Thematic discussions (notes in break-out groups discussions)**

**Research Priority 1: treatment adherence and behaviour change**

| Research aim | Comments |
| --- | --- |
| 1. identify barriers and enablers in adherence to an intervention [e.g. exercise, weight loss] 2. match personality or disease phenotype (non-modifiable) – flag responder and non-responder, adherence and non-adherence 3. identify motivations and barriers in other chronic diseases, are they transferrable to OA? 4. optimal time and type of tailored intervention “top-up” 5. include risk predictors to improve adherence – how to define risk level? 6. evidence for adherence translation into improved outcomes 7. comparative effectiveness of treatment programs and patient and consumer education 8. maintain behavioural change (long-term weight management) post-OA program and sustainability 9. research in a range of ways (e.g. annual review – face to face, phone review, social media networks) 10. test education delivery modes to providers and consumers 11. develop tools & signposting for GPs, pharmacists etc. 12. health services research to increase motivation and decrease barriers 13. explore mechanisms of current practice 14. guideline adherence by practitioners | DISC-(Dominance Influence Steadiness Compliance) - personality profiling  start, initiation & habit creation  personalisation & tailored  patient-centred approach  include psychologists |
| Methods/process (single centre, multi-centre, network collaboration, international links) | Comments |
| - mixed methods:   - observational studies, leading to trials   - qualitative study to look at enablers and barriers to understand how to intervene (who will respond to what) - cluster RCT symptoms outcome and participation/adherence to understand the mechanism/mediator - pragmatic trial   - multi-centre network and each will be slight local modifications - nation-wide, multi-disciplinary (psych, IT, mobile apps, eHealth) - link to private providers with track record and data - need to seek out extant programmes - treat to target strategy (a range of different interventions including behavioural aspects, adherence strategies); once people have been through program those who have lost weight are randomised to adherence strategy follow-up | healthy weight for life program |
| Existing strengths/barriers in Australia | Comments |
| clinical trial networks/workshop (e.g. ANZMUSC)  programs are being rolled out – capacity building is happening now but needs to be monitored |  |
| Potential funding mechanism | Comments |
| local health district networks (leading better value care)  health dept.  Medibank (other insurers)  NHMRC partnership grant  MRFF |  |

**Research Priority 2: disease modification/modifying progression**

| Research aim | Comments |
| --- | --- |
| 1. identify the trajectories (clinical) of patients with OA and the risk factors for each of these trajectories. 2. define disease progression (e.g. definition and measures – symptoms or pathology or both) 3. whole of patient disease not just the local joint OA. Whole patient data versus individual data – example of within subject studies where good correlation between the disease process and symptoms. 4. need to have researchers that understand pain (genomics of pain/symptoms) not unlike other MSK issues. 5. understand symptom progression 6. most valued activity - characterise flare and persistence 7. interaction among structure, function and pain - prediction of characteristics of OA over time 8. role of weight loss / exercise in modifying disease progression 9. better animal / lab / pre-clinical models 10. role of phenotypes in treatment response 11. risk factor indices/predictors of progression 12. identify biomarkers of structural progression within constraints of typically funded trial (could take 5-10 years to address?!) 13. maintain weight loss, slow progression/prevent development of OA 14. use Treat to Target strategy (e.g. achieve certain weight loss by whatever it takes) |  |
| Methods/process (single centre, multi-centre, network collaboration, international links) | Comments |
| - clinical trialists collaborate with basic scientists, including the pain researchers/experts - learn from other MSK researchers - basic science informed by clinical problems – understanding phenotypes - public health campaign by state - cluster randomisation by sporting teams - progression study = no joint replacement, etc. - P = BMI >35 with knee pain – for progression andwithout knee pain – for prevention (substrata – OARSI groupings, malalignment, patello-femoral, etc.) - multi-centre, multi-disciplinary, multi-platform - invite cardiologists, diabetologists on board – link with “healthy eating”, school programs |  |
| Existing strengths in Australia | Comments |
| world leading experts, ANZMUSC, Kolling Institute, suitable research environment to implementation?  pain researchers |  |
| Potential funding mechanism | Comments |
| National Health Medical Research Council research funding  industry sponsors for target modifiers  pain research groups – strict funding for chronic pain mechanism (NSW) |  |

**Research Priority 3: disease prevention**

| Research aim | Comments |
| --- | --- |
| 1. How do we implement an approach to prevent OA through weight loss or injury? (smoking is a several decades issue – similar to OA) 2. does losing weight and maintaining weight prevent prevalence and incidence of OA? 3. community-wide changes – weight control, prevention lens for sport 4. prevention of flare up (pain coping skills) – identify modifiable triggers 5. underlying theme of what is OA – a disease or constellation of symptoms or comorbidities? 6. prevent acute nociceptive pain to more complex chronic pain 7. injury prevention  - implement current Australian effective programmes - Australian Sporting Commission and Schools - roll out to other high-risk populations  1. post traumatic OA (e.g. ACL injury) 2. can we reverse early structural OA?  - matching tissue biomechanical/biochemical environment - lifestyle modification  1. P = young sports people? (focus on one sport?) – prevention study = no pain, normal MRI etc. |  |
| Methods/process (single centre, multi-centre, network collaboration, international links) | Comments |
| - dose-finding study, clustered by sport? - public health campaign – ready yet for famous sports people to support - public health campaign – by state? - cluster randomisation by sports teams - long-term large community-based cluster randomised trials in the field – with outcomes that are routinely collected (population level measures) - either test the different programs FIFA 11+, PEP, KIPP, knee in different sports – or the intervention should be overcoming the many barriers - track a large community cohort longitudinally for some other issues |  |
| Existing strengths in Australia | Comments |
| lots of barriers!  should we work on testing solutions for these? |  |
| Potential funding mechanism | Comments |
| health insurance, accident insurance etc. |  |
